# Supplementary material for: Proton-selective coating enables fast-kinetics high-mass-loading cathodes for sustainable zinc batteries
Source: Nat Commun. 2024 Mar 8;15:2139. doi: 10.1038/s41467-024-46464-9 (PMC10923785; doi:10.1038/s41467-024-46464-9)
Supplement: Supplementary file 1 — Supplementary Information [file 41467_2024_46464_MOESM1_ESM.pdf]

Supplementary Materials

Proton-Selective Coating Enables Fast-Kinetics High-Mass-Loading Cathodes for Sustainable Zinc Batteries

Quanquan Guo[1,2,3,12], Wei Li[1,4,12], Xiaodong Li[1,2,12], Jiaxu Zhang[1], Davood Sabaghi[1], Jianjun Zhang[1], Bowen Zhang[5], Dongqi Li[1], Jingwei Du[1], Xingyuan Chu[1], Sein Chung[6], Kilwon Cho[6], Nguyen Ngan Nguyen[1,2], Zhongquan Liao[5], Zhen Zhang[7], Xinxing Zhang[3], Grégory F. Schneider,[8] Thomas Heine,[9,10,11] Minghao Yu[1]*, Xinliang Feng[1,2]*

[1] Center for Advancing Electronics Dresden (cfaed) & Faculty of Chemistry and Food Chemistry, Technische Universität Dresden, 01062 Dresden, Germany.

[2] Max Planck Institute of Microstructure Physics, Halle (Saale) 06120, Germany.

[3] State Key Laboratory of Polymer Materials Engineering, Polymer Research Institute, Sichuan University, Chengdu 610065, China.

[4] State Key Laboratory of Applied Organic Chemistry, College of Chemistry and Chemical Engineering, Lanzhou University, Lanzhou, 730000, PR China.

[5] Fraunhofer Institute for Ceramic Technologies and System (IKTS), Maria-Reiche-Straße 2, 01109, Dresden, Germany.

[6] Department of Chemical Engineering, Pohang University of Science and Technology, Pohang, 37673 South Korea.

[7] School of Chemistry and Materials Science, University of Science and Technology of China, 230026 Hefei, China.

[8] Leiden Institute of Chemistry, Leiden University, P.O. Box 9502, Leiden 2300 RA, The Netherlands

[9] Theoretical Chemistry, Technische Universität Dresden, 01062 Dresden, Germany.

[10] Institute of Resource Ecology, Helmholtz-Zentrum Dresden-Rossendorf, Leipzig Research Branch, 04316 Leipzig, Germany.

[11] Department of Chemistry, Yonsei University, Seodaemun-gu Seoul 120-749, Korea.

[12] These authors contributed equally: Quanquan Guo, Wei Li, Xiaodong Li

* Email: minghao.yu@tu-dresden.de; xinliang.feng@tu-dresden.de

Supplementary Figures

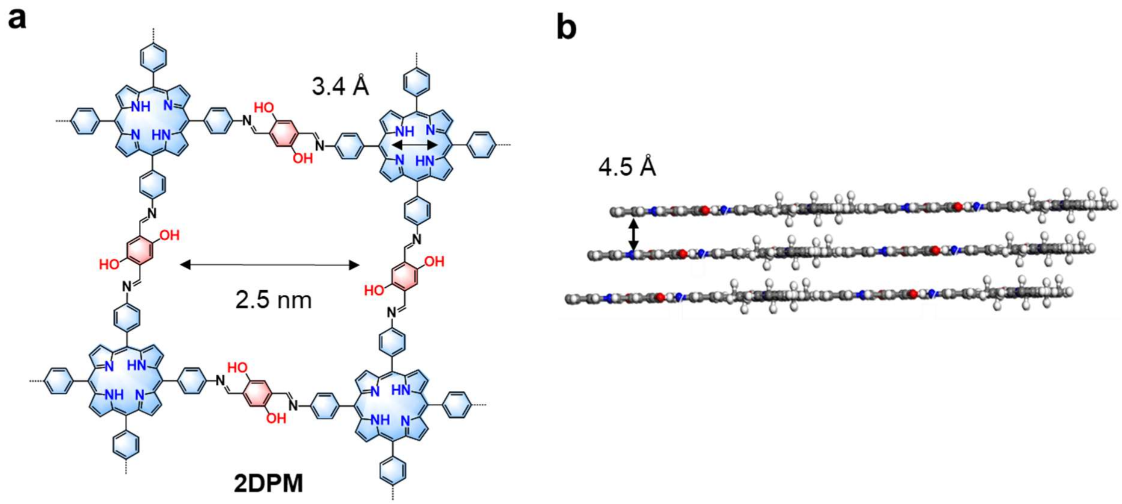

Supplementary Fig. 1. Chemical structure of two-dimensional polyimine membrane (2DPM). a The chemical structure of 2DPM nanomembrane with 2.5 nm imine-enclosed pore and 3.4 Å central porphyrin pore. **b** The inclined AA stacking of 2DPM with an interlayer distance of 4.5 Å (white atoms: H, gray atoms: C, red atoms: O, blue atoms: N).

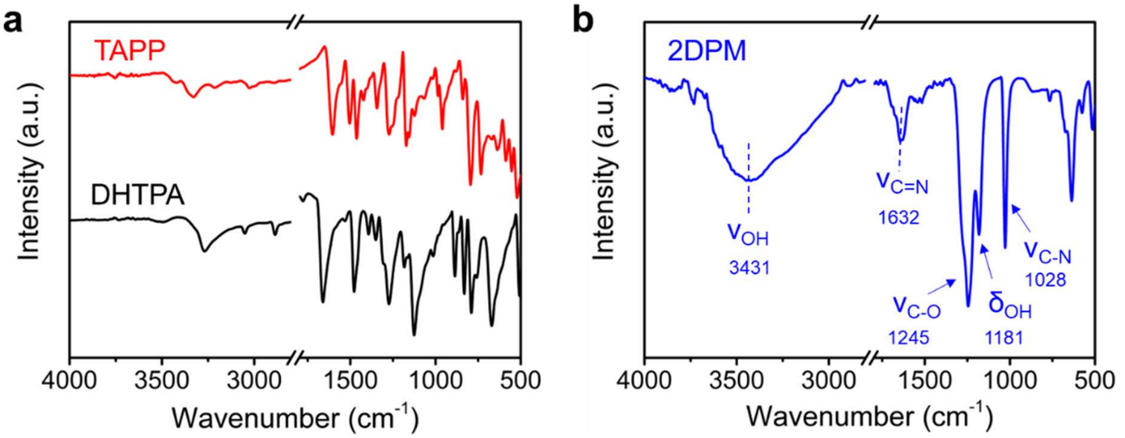

Supplementary Fig. 2. Fourier-transform infrared (FTIR) spectra of monomers and 2DPM. FTIR spectra of **a** the 5, 10, 15, 20-tetrakis (4-aminophenyl) porphyrin (TAPP) monomer, 5-dihydroxyterephthalaldehyde (DHTPA) monomer, and **b** 2DPM. FTIR spectrum of 2DPM demonstrates the formation of imine bond (1632 cm^{-1}, $\nu_{C=N}$) and the presence of hydroxyl (3431 cm^{-1}, ν_{OH})[1].

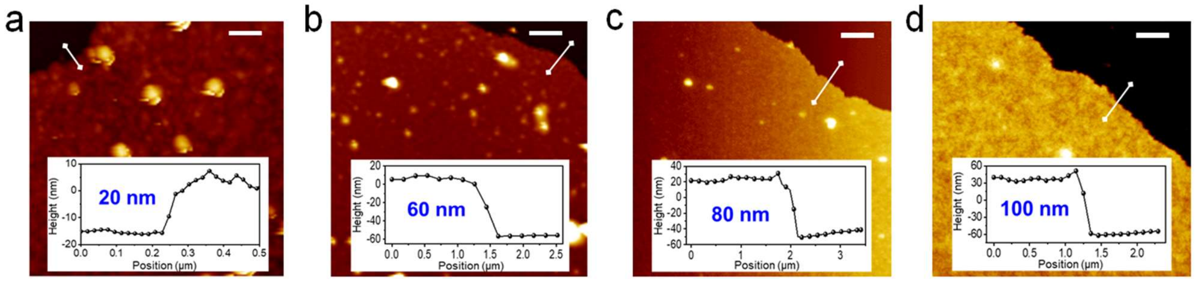

Supplementary Fig. 3. Atomic force microscopy (AFM) images of 2DPM with different thicknesses. 2DPM with a thickness of **a** 20 nm, **b** 60 nm, **c** 80 nm, and **d** 100 nm. Scale bars: 2 μm. The insets show the height profile along the white lines indicated in the AFM images.

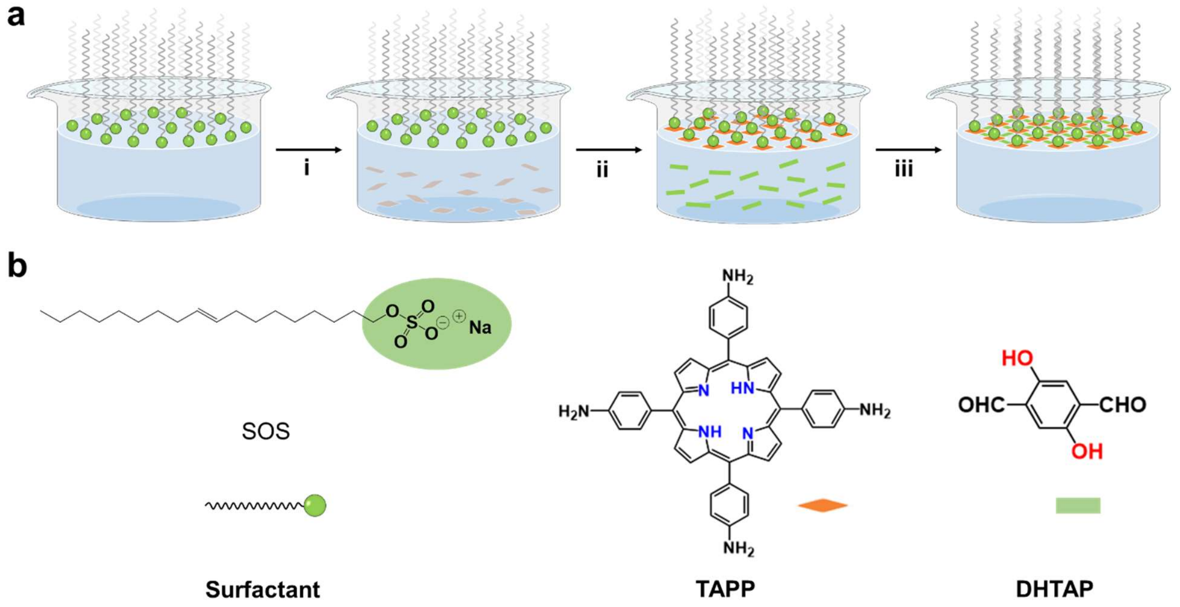

Supplementary Fig. 4. Schematic diagram for surfactant-monolayer-assisted interfacial synthesis (SMAIS) method. a The classic SMAIS at air-water interface[2]. (**i**) Surfactant (sodium oleyl sulfate, SOS) self-assembles into a monolayer with SO_4^{2-} polar head groups toward the water phase. SOS is able to direct the pre-assembling of precursors at the interface and facilitate the Schiff-base polycondensation reaction between TAPP and DHTAP monomers. (**ii**) Due to hydrogen bonding and electrostatic interactions, the protonated TAPP is readily adsorbed underneath the SOS monolayer. In the next step, DHTAP diffuses to the air/water interface and triggers the Schiff-base condensation reaction between amine and aldehyde groups. (**iii**) Benefitting from the dynamic characteristic of imine linkage, 2DPM self-corrects the polymer chain arrangement by molecular exchange under a thermodynamic equilibrium state, enabling a long-range ordered 2D polymer network. The intramolecular hydrogen formed at the imine center and strong π-π interactions between porphyrin monomers can efficiently promote the crystallinity, structural rigidity, and chemical stability of the resultant nanomembrane. After keeping the reaction for 5 days at 50 ℃, a large-area, ultrathin, crystalline, and homogeneous 2DPM membrane is successfully prepared. **b** Chemical structures of SOS, TAPP, and DHTAP.

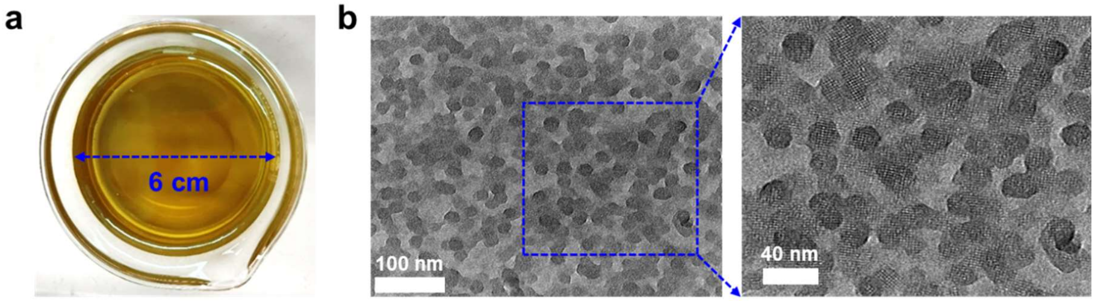

Supplementary Fig. 5. Morphology characterization of 2DPM. a Digital photo of the synthesized 2DPM with a large area of 28 cm^2. **b** High-resolution transmission electron microscopy (HR-TEM) images of 2DPM revealing its high crystallinity and domain sizes ranging from 10 to 50 nm.

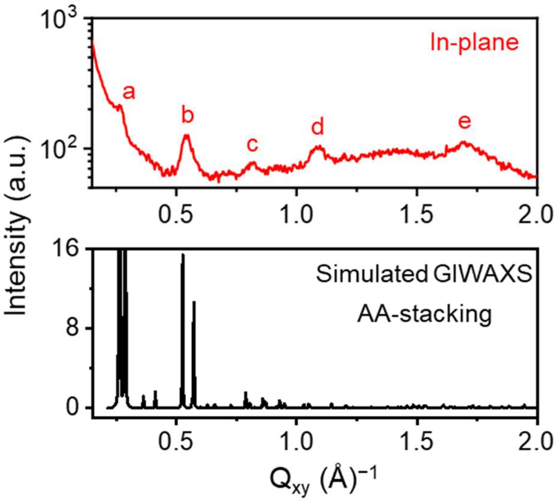

	hkl	Q_{xy} (Å)$^{-1}$
a	(010), (100)	0.27
b	(020), (120), (200)	0.54
c	(030), (130), (300)	0.82
d	(040), (140), (410), (400)	1.09
e	(511), (540),(600), (620), (611)	1.69

Supplementary Fig. 6. In-plane grazing incidence wide-angle X-ray scattering (GIWAXS) pattern. In-plane GIWAXS pattern of 2DPM and simulated GIWAXS pattern based on an inclined AA-stacking model.

The GIWAXS measurement was performed to investigate the macroscopic crystallinity and lattice structure of 2DPM (Fig. 1c). The in-plane peaks at 0.25 Å^{-1} and 0.5 Å^{-1} are assigned to the Bragg reflections of a square lattice (100) and (200) with $a = b = 25.3$ Å. In the vertical direction, 2DPM shows a much lower out-plane crystallinity degree, which is evidenced by the faint scattering peaks along the Q_Z axis. An intense arc signal at $Q_Z \approx 1.6$ Å^{-1} corresponds to the interlayer π–π stacking with a spacing of ~ 4.2 Å[2]. These results match well with an inclined AA-stacking model simulated through DFT calculations, as revealed in Supplementary Fig. 1 and 6.

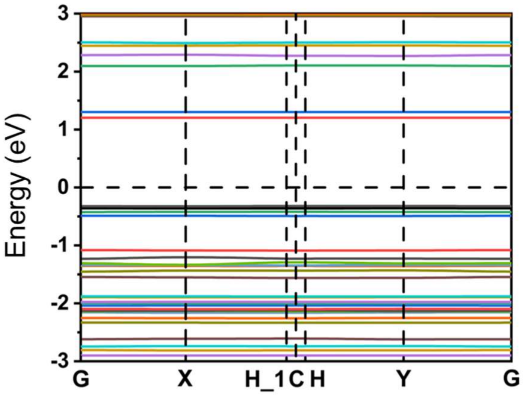

Supplementary Fig. 7. The simulated electronic band structure of 2DPM.

A giant band gap of 1.53 eV is identified for 2DPM, suggesting its poor electron-conductive nature. Besides, the electron conductivity of 2DPM was experimentally assessed through a four-point probe measurement. The sheet resistance of 2DPM-80 reaches up to 6.1×10^7 Ohm sq^{-1}.

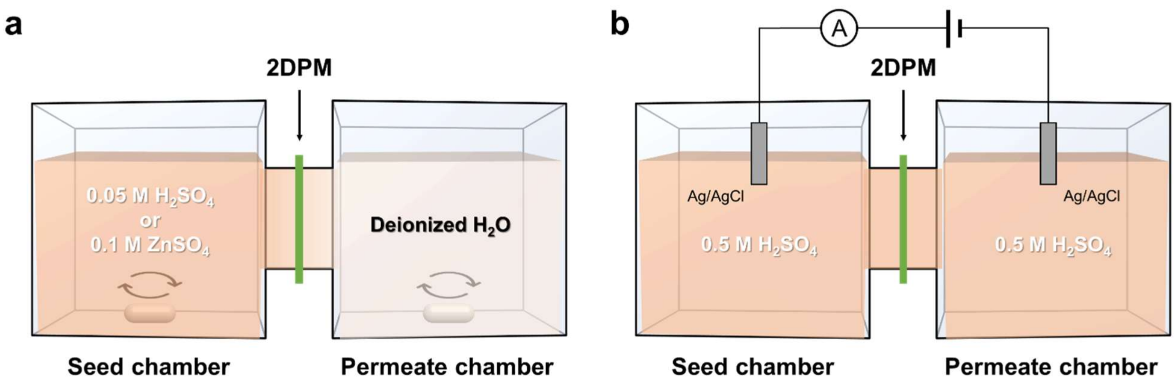

Supplementary Fig. 8. Ion permeation test setups. a The setups used for the concentration-driven ion permeation and **b** electric conductance measurements[3].

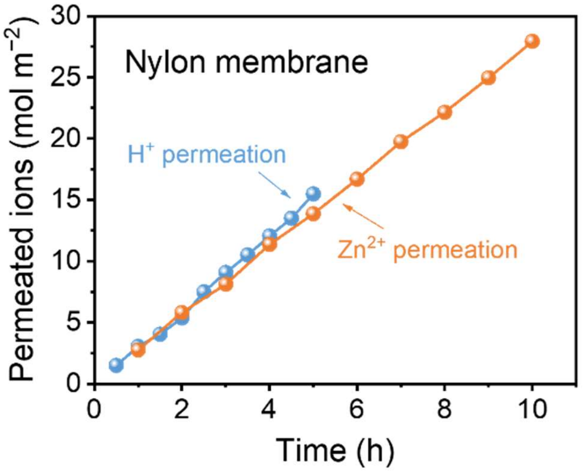

Supplementary Fig. 9. H^+ and Zn^{2+} permeation curves of the Nylon microporous membrane (pore size of 0.45 μm). The Nylon membrane shows ultrahigh ion flux and almost the same permeation rate for H^+ (3.01 mol m^{-2} h^{-1}) and Zn^{2+} ions (2.82 mol m^{-2} h^{-1}), suggesting the negligible transport selectivity for H^+ and Zn^{2+}.

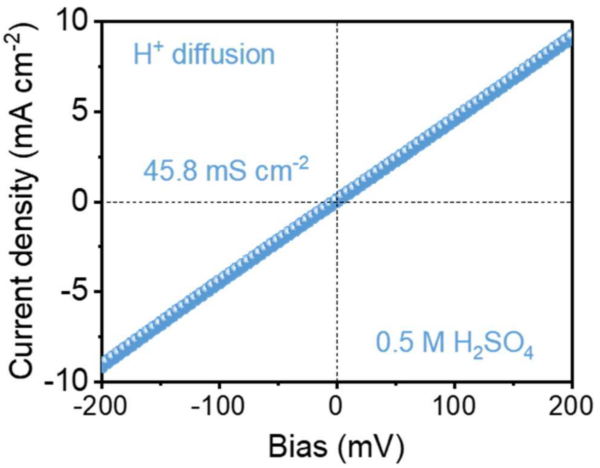

Supplementary Fig. 10. Electric field-driven permeation test of 2DPM-80. Current density (I) as a function of applied voltage (V) for 2DPM-80 in 0.5 M H_2SO_4. The areal ion conductivity (σ) was determined by the equation σ = I/V.[4] According to the linear I-V response, the areal conductivity of 2DPM-80 was determined to be 45.8 mS cm^{-2}.

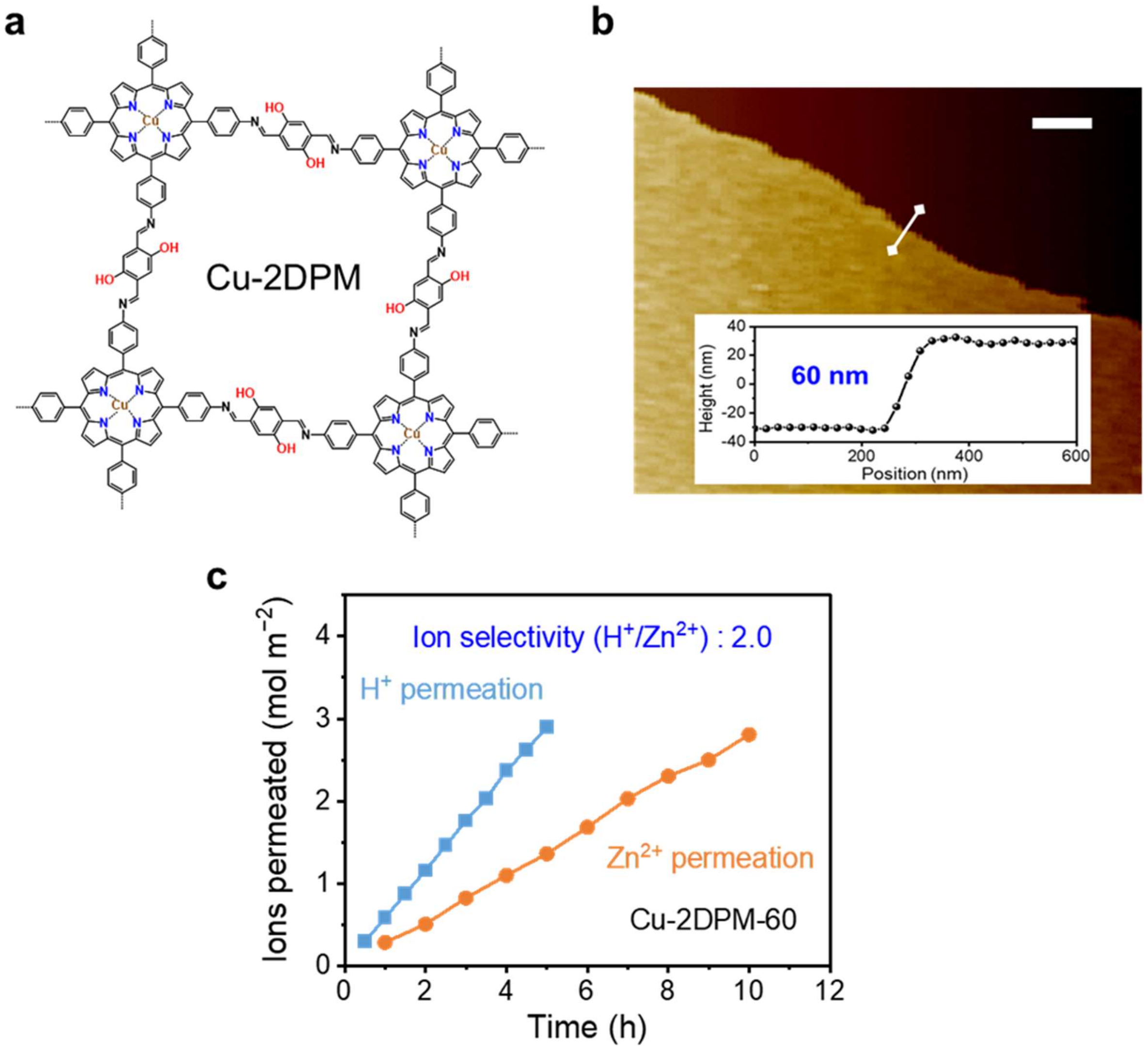

Supplementary Fig. 11. Chemical structure, AFM image, and ion permeation behavior of Cu-2DPM-60. **a** Chemical structure of Cu-2DPM-60. **b** AFM image of Cu-2DPM-60. Scale bar: 2 μm. The inset shows the height profile along the white line indicated in the AFM image. **c** The H^+ and Zn^{2+} ion permeation curves of Cu-2DPM-60.

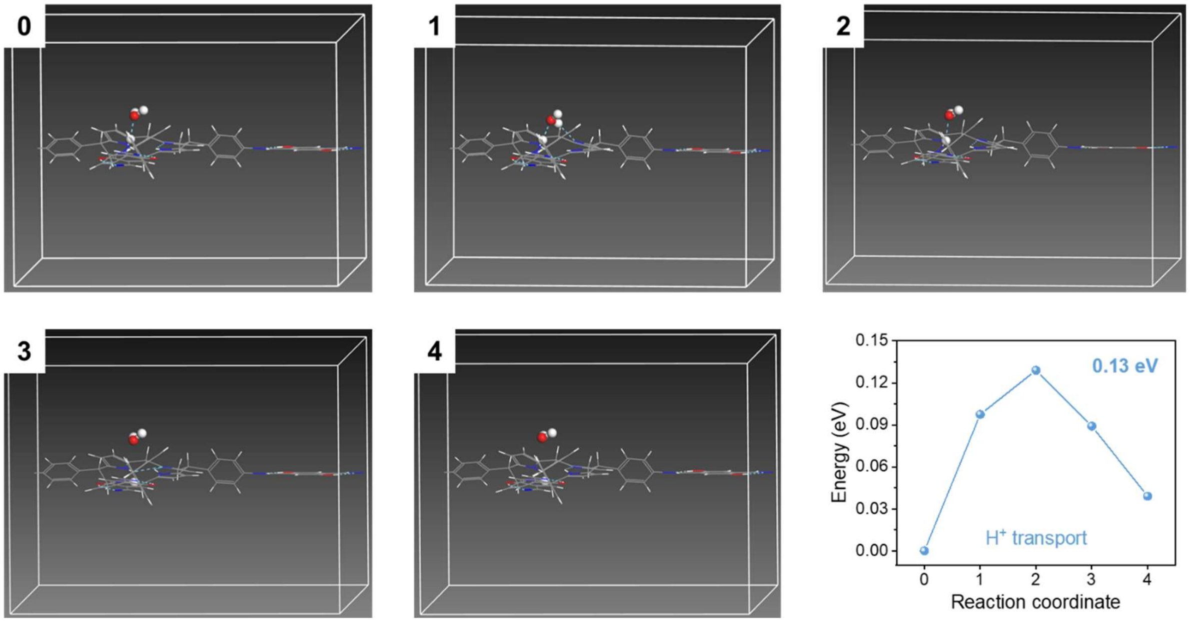

Supplementary Fig. 12. Density functional theory (DFT) simulation for proton passing through porphyrin center. The simulated H^+ transport path and the corresponding energy profiles through the porphyrin center (white atoms: H, gray atoms: C, red atoms: O, blue atoms: N). Owing to the size effect, the hydrated proton tent to desolvation first and then pass through the central porphyrin pore. The calculated energy barrier for H^+ transport is 0.13 eV.

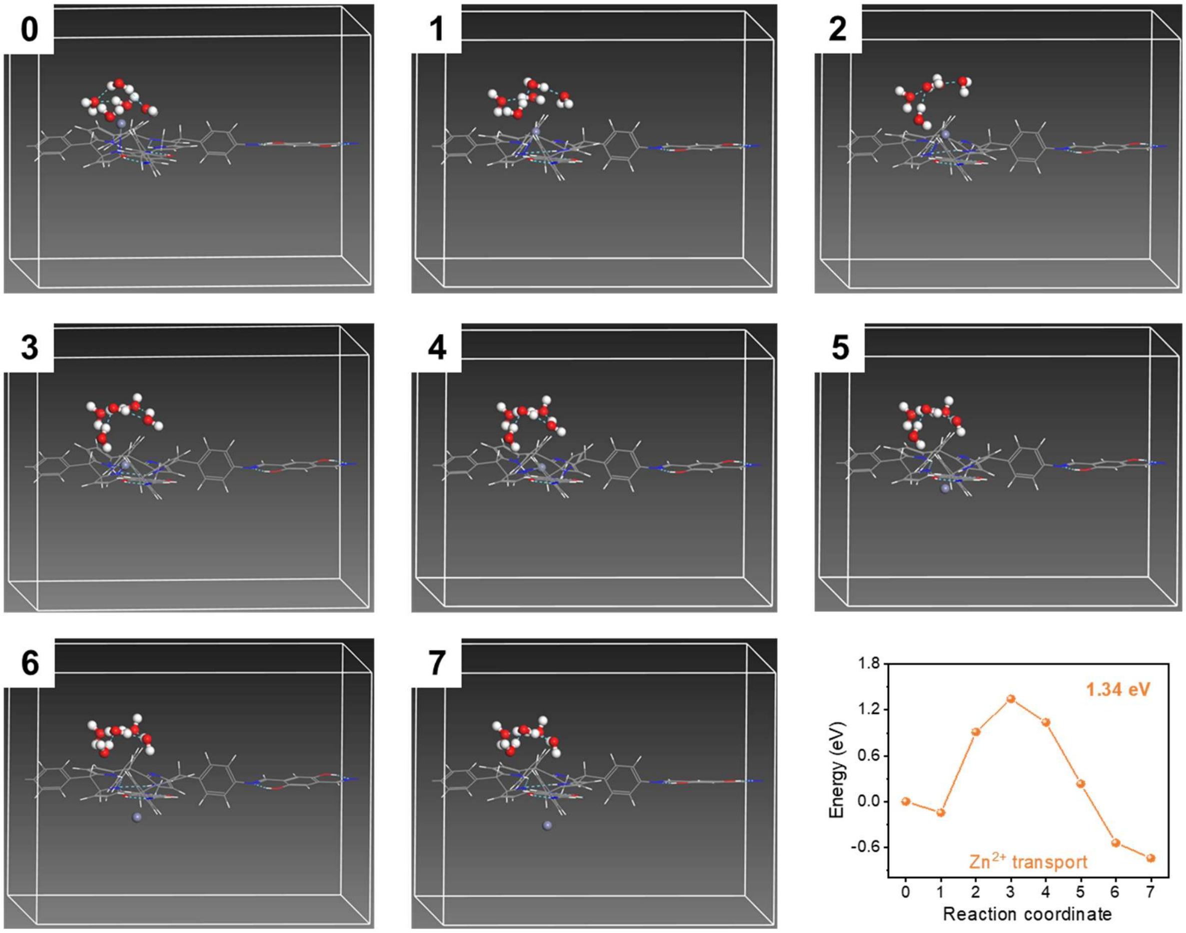

Supplementary Fig. 13. DFT simulation for zinc ions passing through porphyrin center. The simulated Zn^{2+} transport path and the corresponding energy profiles through the porphyrin center (white atoms: H, gray atoms: C, red atoms: O, blue atoms: N). After desolvation, the Zn^{2+} could pass through the porphyrin with a high energy barrier of 1.34 eV.

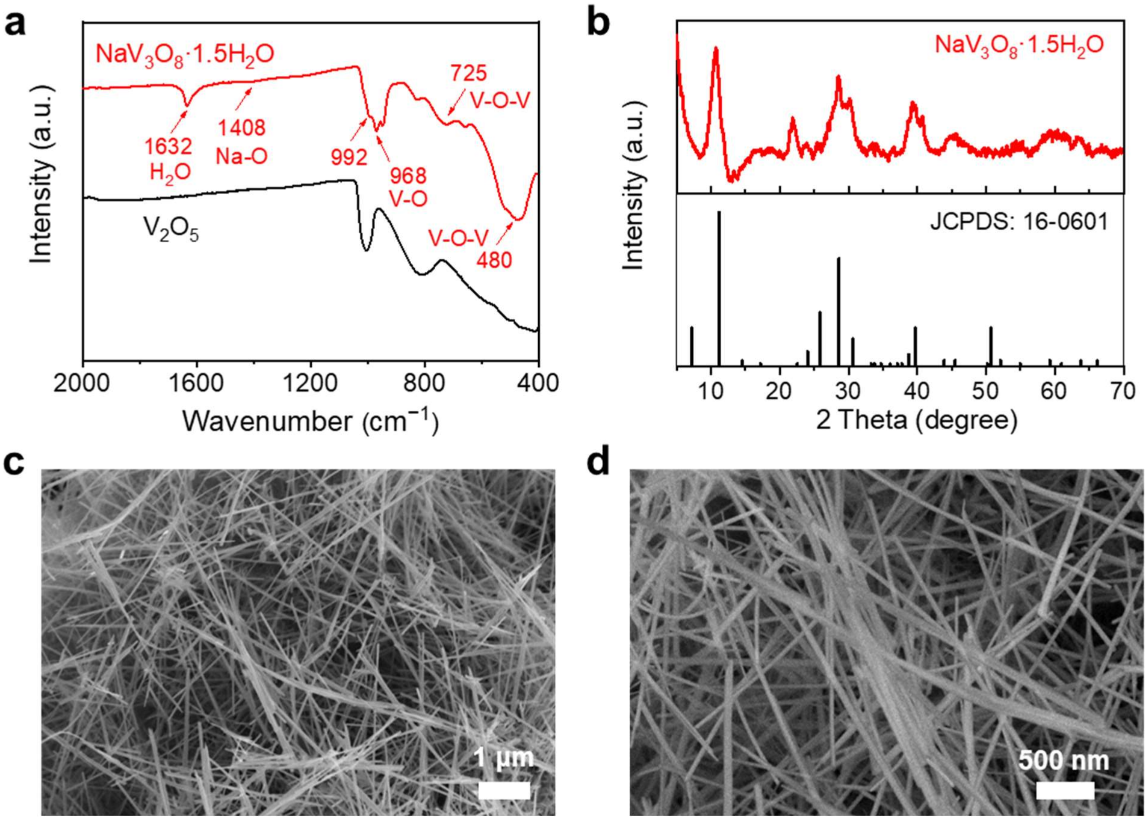

Supplementary Fig. 14. Structural characterizations of $NaV_3O_8 \cdot 1.5H_2O$ (NVO) nanowires. a FTIR spectra, **b** X-ray diffraction (XRD) pattern and **c**, **d** scanning electron microscopy (SEM) images of $NaV_3O_8 \cdot 1.5H_2O$ nanowires at different magnifications. The FTIR peaks at 1632, 968, and 480 cm^{-1} are assigned to H_2O, $\nu_{(V\text{-}O)}$, and $\nu_{(V\text{-}O\text{-}V)}$, respectively. In the XRD pattern of NVO, its characteristic peaks are in good agreement with the $P2_1/m$ space group (JCPDS no. 16–0601)[5].

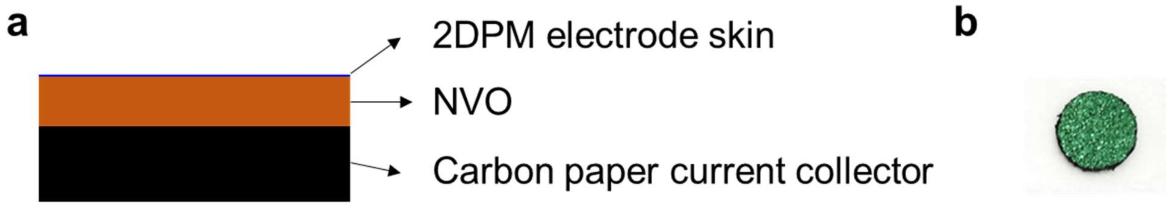

Supplementary Fig. 15. Scheme and photo of 2DPM-covered NVO. a Schematic illustration and **b** digital photo 2DPM-covered NVO.

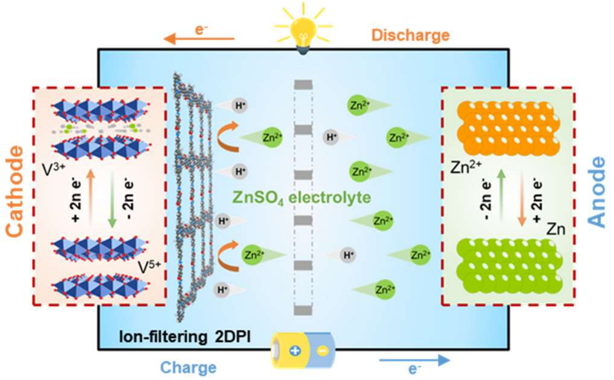

Supplementary Fig. 16. Schematic illustration for the assembled aqueous zinc battery. Schematic illustration showing the working mechanism of the assembled AZB with the 2DPM electrode skin (gray spheres: H^+, green spheres: Zn^{2+}, blue spheres: V, red spheres: O).

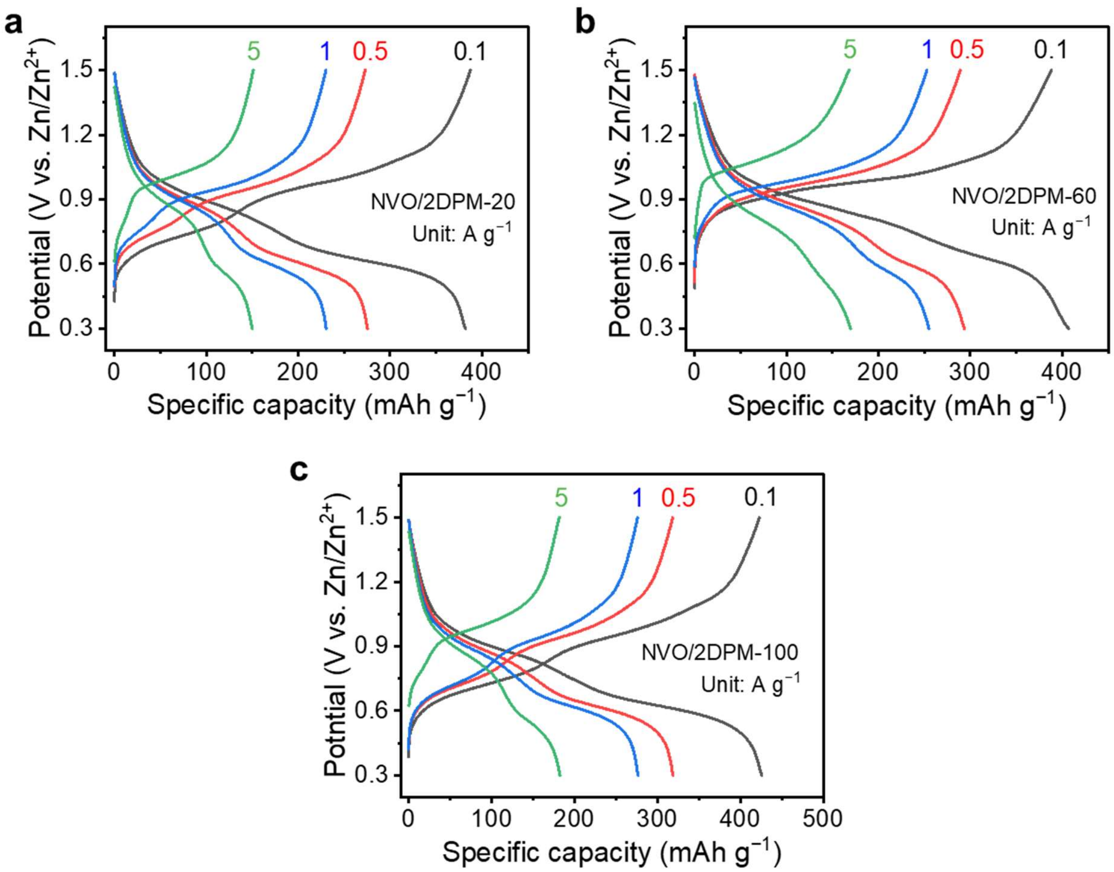

Supplementary Fig. 17. Galvanostatic charge-discharge (GCD) profiles of NVO/2DPM. GCD profiles of **a** NVO/2DPM-20, **b** NVO/2DPM-60, and **c** NVO/2DPM-100 at different current densities.

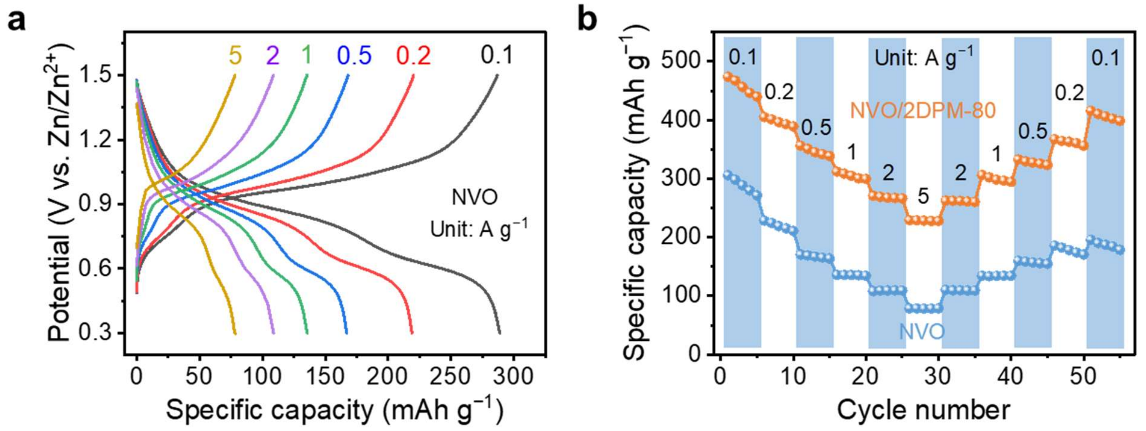

Supplementary Fig. 18. Electrochemical performance of NVO and NVO/2DPM-80. a GCD curves of NVO at different current densities. **b** Rate performance of NVO and NVO/2DPM-80.

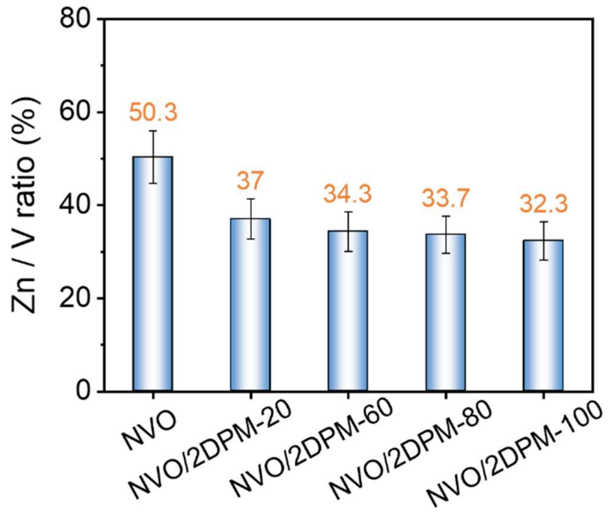

Supplementary Fig. 19. The Zn/V ratios of the fully discharged NVO covered by 2DPM with different thicknesses. The error bars represent the standard deviation calculated from five parallel tests.

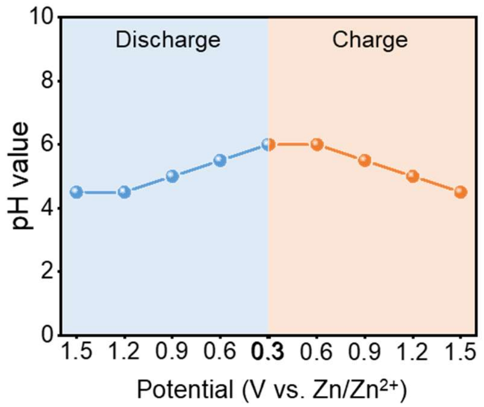

Supplementary Fig. 20. The pH evolution in the electrolyte during one discharge/charge cycle of the NVO/2DPM electrode.

It is essential to note that the water solvent in the electrolyte can act as a proton reservoir. The consumption of H^+ in the electrolyte would disrupt the equilibrium of the hydrolysis reaction of Zn^{2+} (equation (1)), triggering the generation of more H^+ charge carriers for the cathode. We evaluated the electrolyte pH evolution during a discharge/charge cycle in a 2-electrode Swagelok cell. Due to the limited amount of the used electrolyte, we were not able to test the pH with a standard pH meter. Instead, pH paper strips were employed to assess the pH variation (Supplementary Fig. 20). As expected, the insertion of H^+ into the cathode causes a slight pH increase within a range of 4~6.

$$[Zn(H_2O)_6]^{2+} + H_2O \leftrightarrow [Zn(H_2O)_{6-x}(OH)]^{(2-x)+} + xH^+ \quad (1)$$

Moreover, we should note that the H^+-involved cathode reaction could lead to changes in the electrolyte environment, accelerating the parasitic reactions of the Zn metal anode. Specifically, the involvement of H^+ charge carriers was identified as a crucial reason for the formation of the passivation layer (i.e., $Zn_4SO_4(OH)_6{\cdot}4H_2O$) on the Zn anode[5]. However, considering the substantial benefits of H^+ charge carriers brought for the cathode, such as fast reaction kinetics, high mass loading, and large areal capacity, we believe that H^+ can be considered suitable charge carriers for cathodes. Certainly, particular attention should also be paid to protecting Zn metal anodes when full devices are assembled for practical applications. To this end, a range of previously reported strategies could be adopted, such as interphase construction and electrolyte additives with pH-adaptive capability[6-8].

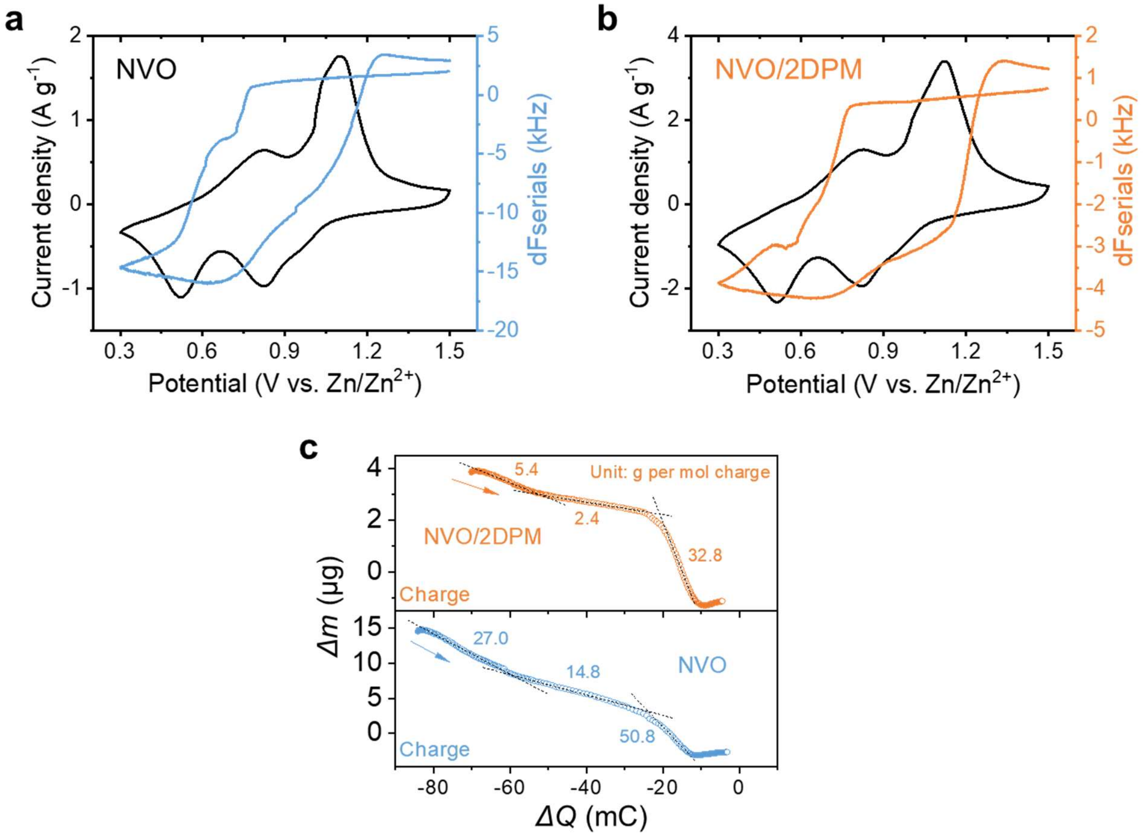

Supplementary Fig. 21. Cyclic voltammetry (CV) curves and the corresponding electrochemical quartz crystal microbalance (EQCM) frequency response. CV curves and EQCM frequency response of **a** NVO and **b** 2DPM-80-covered NVO. The frequency change could be transformed to mass change according to the Sauerbrey equation[9]. **c** Δm as a function of ΔQ for NVO and 2DPM-80-covered NVO in the EQCM measurement during charge.

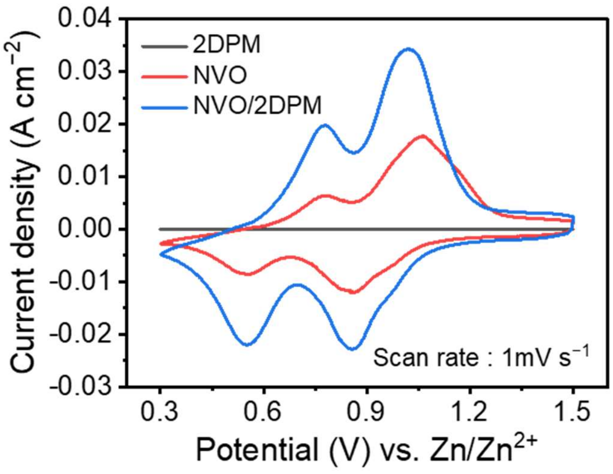

Supplementary Fig. 22. **CV curves of pristine 2DPM-80, NVO, and NVO/2DPM at 1 mV s^{-1}.**

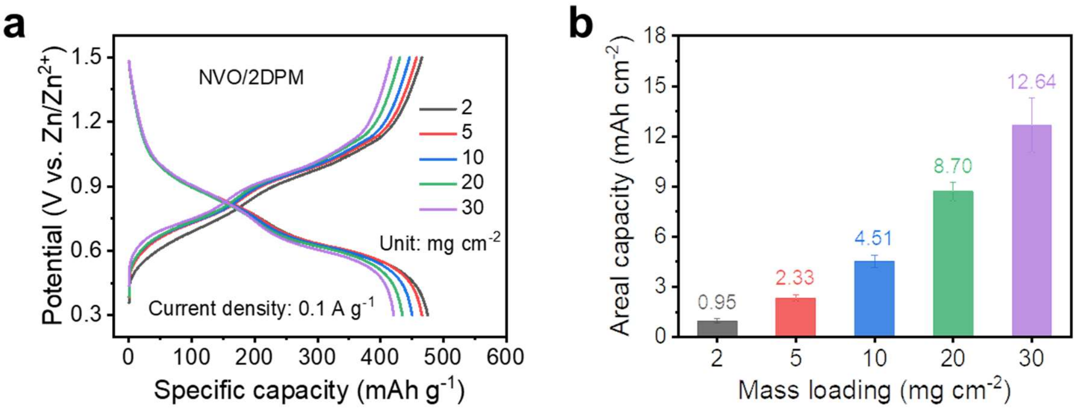

Supplementary Fig. 23. The measured capacities of NVO/2DPM electrodes with different mass loading. a GCD curves of NVO/2DPM electrodes with different mass loadings. **b** Corresponding areal capacity variation as a function of mass loading. The error bars represent the standard deviation calculated from five parallel tests.

NVO/2DPM electrodes were fabricated with different mass loadings (i.e., 2 ± 0.5, 5 ± 1, 10 ± 2, 20 ± 2, and 30 ± 3 mg cm^{-2}). Supplementary Fig. 23a compares their GCD curves at a current density of 0.1 A g^{-1}. Along with the mass loading increase from 2 to 30 mg cm^{-2}, the specific capacity slightly drops from 475.4 to 421.2 mAh g^{-1}. Meanwhile, the areal capacity experiences a significant enhancement from 0.95 mAh cm^{-2} to an ultrahigh value of 12.64 mAh cm^{-2} (Supplementary Fig. 23b). This result further highlights the effective role of the 2DPM coating in boosting the charge-storage kinetics of high-mass-loading cathodes for aqueous Zn batteries.

Supplementary Table. S1. Performance comparison of AZB cathodes.

	Mass loading (mg cm^{-2})	Specific capacity (mAh g^{-1})	Areal capacity (mAh cm^{-2})	Energy density (Wh kg^{-1})	Areal energy density (Wh m^{-2})	Ref
$NaV_3O_8\cdot 1.5H_2O$	2	288.8	0.6	235.11	4.7	5
LiV_3O_8	-	172	-	225	-	10
$Ca_{0.25}V_2O_5\cdot nH_2O$	5.7	340	1.9	267	15.2	11
$Zn_{0.25}V_2O_5\cdot nH_2O$	5-7	300	2.1	260	18.2	12
$Zn_3V_2O_7(OH)_2\cdot 2H_2O$	4-5	213	1.1	214	10.7	13
δ-$Ni_{0.25}V_2O_5\cdot nH_2O$	2.5-3.5	402	1.4	286.6	10	14
$VOPO_4$	2	139	0.3	217	4.3	15
α-MnO_2	1-5	285	1.4	170	8.5	16
β-MnO_2	2	225	0.5	75.2	1.5	17
PANI-intercalated MnO_2	2	298	0.6	-	-	18
$CaMnO_4$	-	250	-	280	-	19
$ZnMn_2O_4$	2	150	0.3	202	4	20
α-MoO_3	0.6	164.9	0.1	96	0.6	21
Co-intercalated MoO_3	2.5	356.8	0.9	198	5	22
MoS_2	2	202.6	0.4	148.2	3	23
$Zn_3[Fe(CN)_6]_2$	8	73.7	0.6	100	8	24
Co–Mn Prussian Blue Analog	0.8-1	128.6	0.1	180	1.8	25
NVO/2DPM	10	450.5	4.5	338.3	33.8	This work
ε-MnO_2/2DPM	5	280.3	1.4	367.1	18.4	This work
α-MoO_3/2DPM	10	310	3.1	191.1	19.1	This work

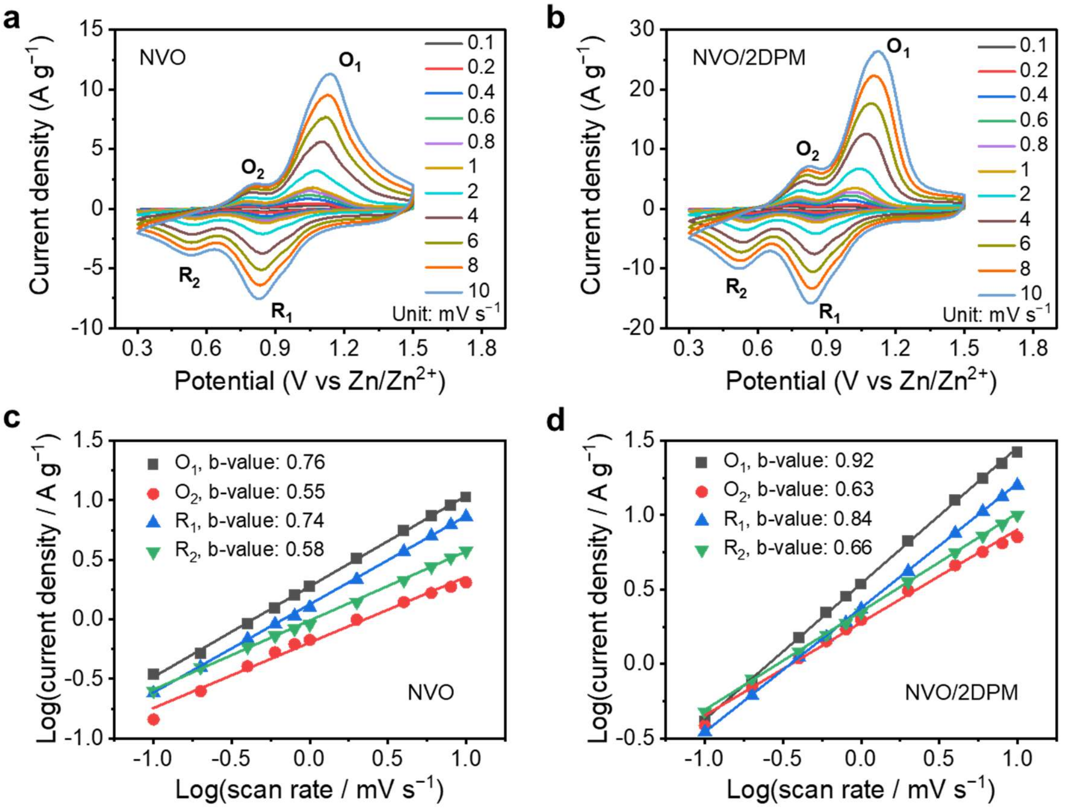

Supplementary Fig. 24. Charge-storage kinetics analysis of NVO and NVO/2DPM electrodes. CV curves of **a** NVO and **b** NVO/2DPM at various scan rates. b-values of **c** NVO and **d** NVO/2DPM at two pairs of redox peaks.

The charge-storage kinetics analysis of NVO and NVO/2DPM was further performed by collecting their CV profiles at scan rates ranging from 0.1 to 10 mV s^{-1} (Supplementary Fig. 24a and b). Consistent with the GCD profiles, two-step redox reactions can be identified by the two pairs of redox peaks at 0.65 ~ 1.38 V vs. Zn/Zn^{2+} (denoted O_1/R_1) and 0.3 ~ 0.88 V vs. Zn/Zn^{2+} (denoted O_2/R_2). The classic equation (2) was adopted to describe the relationship between peak current density (I, A g^{-1}) and the sweep rate (v, mV s^{-1}). The b-value in the equation characterizes the kinetics of the redox reaction, with a value of 0.5 indicating sluggish diffusion-controlled behavior, and a value of 1.0 indicating rapid surface-controlled behavior[21]. By plotting log (I) as a function of log (v), it is observed that all the redox peaks of NVO/2DPM display higher b-values than NVO (Supplementary Fig. 24c and d). In detail, b-values for O_1, R_1, O_2, and R_2 are 0.92, 0,84, 0.63, and 0.66 for NVO/2DPM, and 0.76, 0.55, 0.74, and 0.58 for NVO.

$$I = av^b \qquad (2)$$

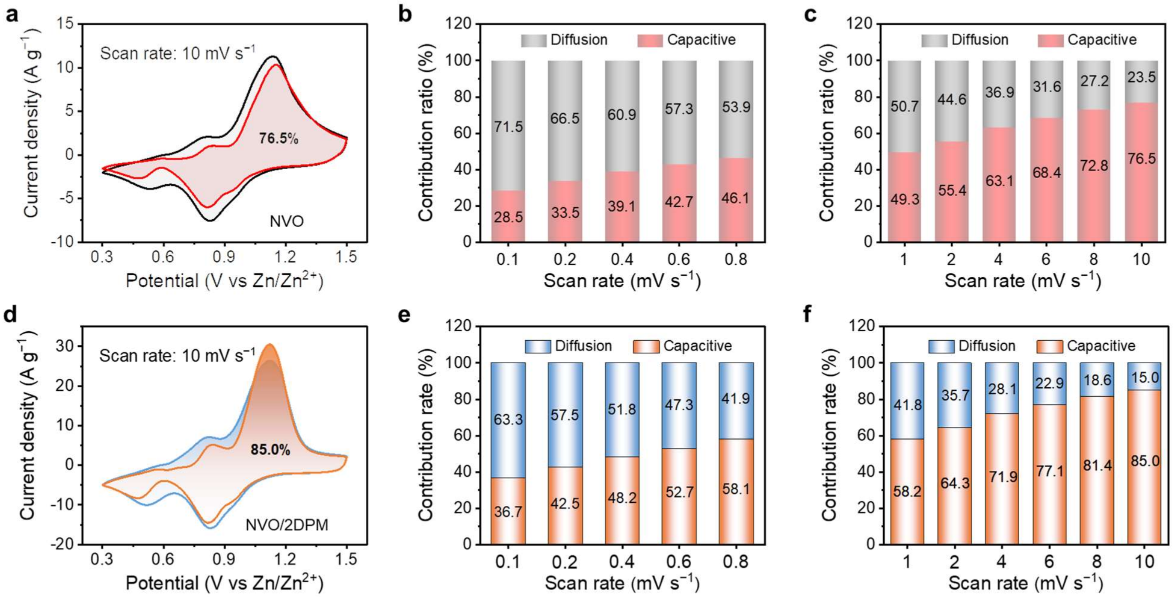

Supplementary Fig. 25. Capacitive and diffusion-controlled contribution analysis for NVO and d NVO/2DPM electrodes. CV curve at 10 mV s^{-1} of **a** NVO and **d** NVO/2DPM with separation between the total current and the capacitive current. Normalized contribution ratios of the capacitive and diffusion-controlled currents as a function of scan rate for **b, c** NVO and **e, f** NVO/2DPM.

Likely, the quantification of the capacitive contribution (k_1v) and diffusion-controlled contribution ($k_2v^{1/2}$) to the overall charge storage, according to equation (3)[26], supports the boosted charge-storage kinetics of NVO/2DPM (Supplementary Fig. 25). The capacitive contribution of NVO/2DPM continuously increases along with the increase of the scan rate from 0.1 to 10 mV s^{-1}, achieving from 36.7% at 0.1 mV s^{-1} to a maximum value of 85.0% at 10 mV s^{-1}. These fraction values are substantially higher than those of NVO.

$$I = k_1v + k_2v^{1/2} \quad (3)$$

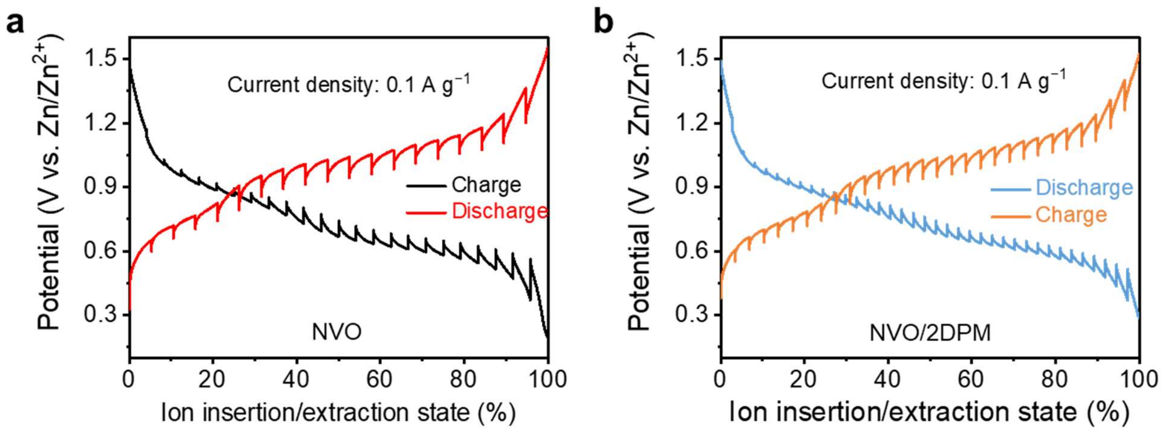

Supplementary Fig. 26. Galvanostatic intermittent titration technique (GITT) charge/discharge curves. GITT charge/discharge curves of **a** NVO and **b** NVO-2DPM. The electrodes were charged/discharged at 0.1 A g^{-1} for 10 min and rest for 1 h.

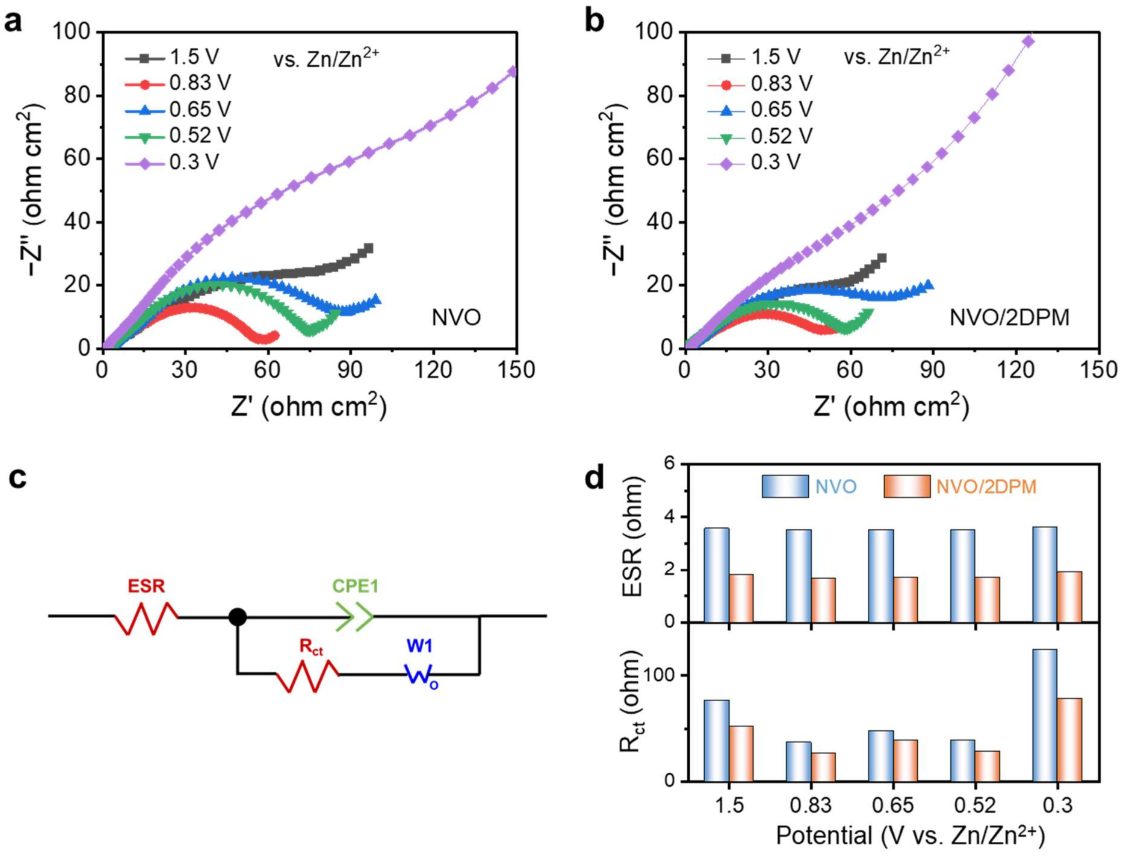

Supplementary Fig. 27. Electrochemical impedance spectroscopy (EIS) analysis for NVO and NVO/2DPM electrodes. Nyquist plots of **a** NVO and **b** NVO/2DPM at different potentials. **c** A equivalent circuit model used for the EIS data fitting. **d** *ESR* and R_{ct} at different potentials of NVO and NVO/2DPM.

To evaluate the effect of 2DPM on the internal resistance, the EIS measurement was performed for NVO and NVO/2DPM at different potentials (Supplementary Fig. 27a-b). By fitting the EIS results with an equivalent circuit shown in Supplementary Fig. 27c, the equivalent series resistance (*ESR*) and charge-transfer resistance (R_{ct}) are determined for both electrodes. Typically, ESR reflects the internal or Ohmic resistance of the whole electrochemical system. As revealed in Supplementary Fig. 27d, *ESR* of NVO/2DPM is slightly lower than NVO, indicating that the 2DPM coating could slightly alleviate the interfacial contact resistance, instead of impeding the electronic conduction. More importantly, NVO/2DPM presents significantly lower R_{ct} than NVO, manifesting the enhanced charge-transfer efficiency of NVO/2DPM associated with the enriched H^+ as charge carriers.

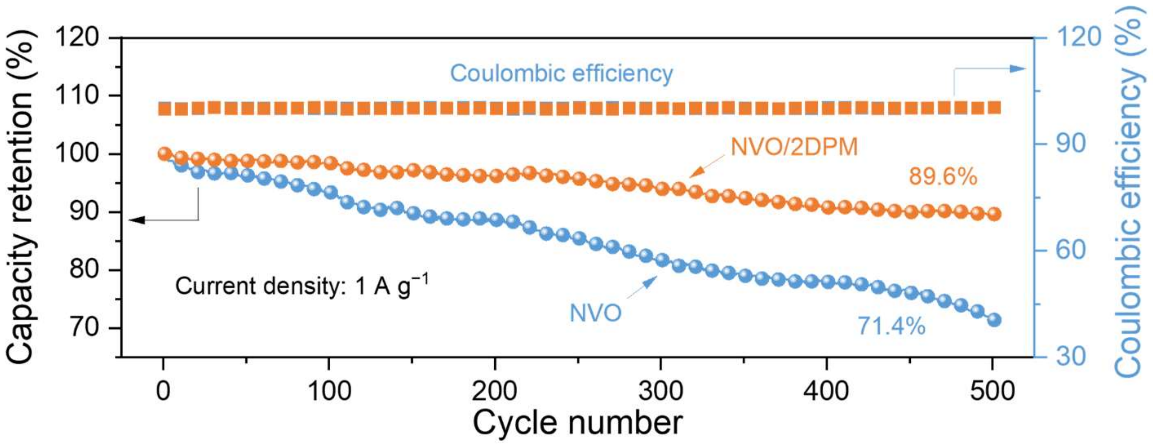

Supplementary Fig. 28. Electrochemical performances of NVO/2DPM. Cycling performance and coulombic efficiency of NVO and NVO/2DPM at 1 A g^{-1}.

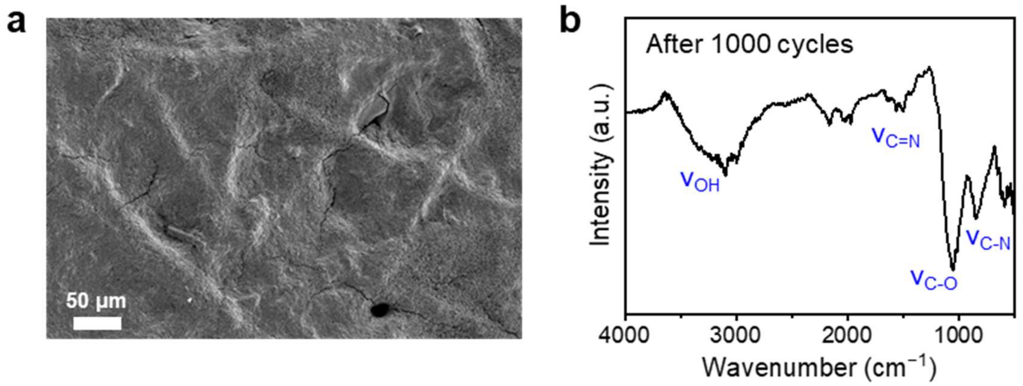

Supplementary Fig. 29. Structural characterizations of NVO/2DPM electrode after cycling. a SEM image and **b** FTIR spectrum of NVO/2DPM after charging and discharging for 1,000 cycles in 2 M $ZnSO_4$. After the cycling test, the 2DPM still closely covers the NVO surface and maintains the characteristic FTIR peaks including $\nu_{C=N}$, ν_{OH}, ν_{C-O}, and ν_{C-N}. All the FTIR peaks shift to lower wavenumber, which is ascribed to the absorption of $ZnSO_4$ on the 2DPM.

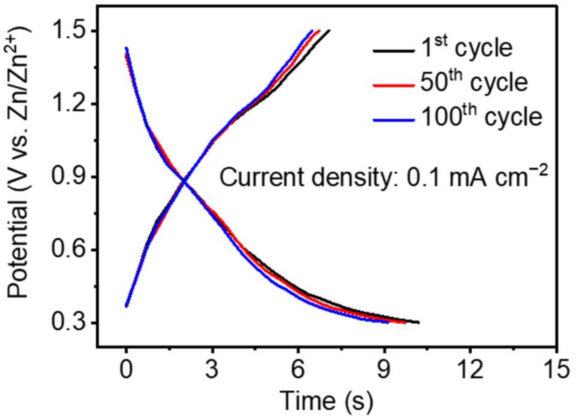

Supplementary Fig. 30. Electrochemical stability of 2DPM. The 1st-cycle, 50th-cycle, and 100th-cycle GCD curves of 2DPM-80 at 0.1 mA cm^{-2}.

We conducted a GCD measurement of a 2DPM-80 electrode (2DPM-80-covered carbon paper) in a 2-electrode Swagelok cell, employing Zn foil as the counter electrode and 2 M $ZnSO_4$ as the electrolyte. As depicted in Supplementary Fig. 30, 2DPM exhibits nearly identical GCD profiles, further verifying its excellent electrochemical stability within the potential range of 0.3~1.5 V vs. Zn/Zn^{2+}.

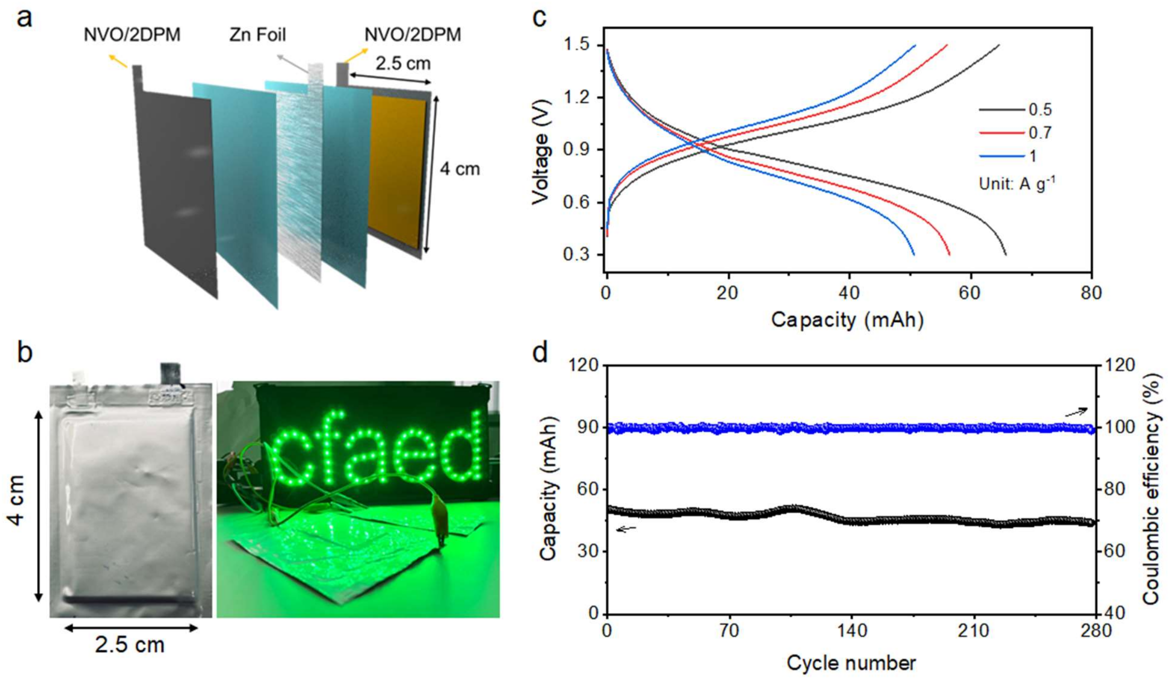

Supplementary Fig. 31. Demonstration of the AZB pouch cell. a Schematic illustration showing the "sandwich" configuration of the pouch cell. **b** Digital photos of a pouch cell and a LED board powered by two pouch cells connected in series. **c** GCD curves of the pouch cell at 0.5, 0.7, and 1 A g^{-1}. **d** Cyclic performance of the pouch cell at 1 A g^{-1}.

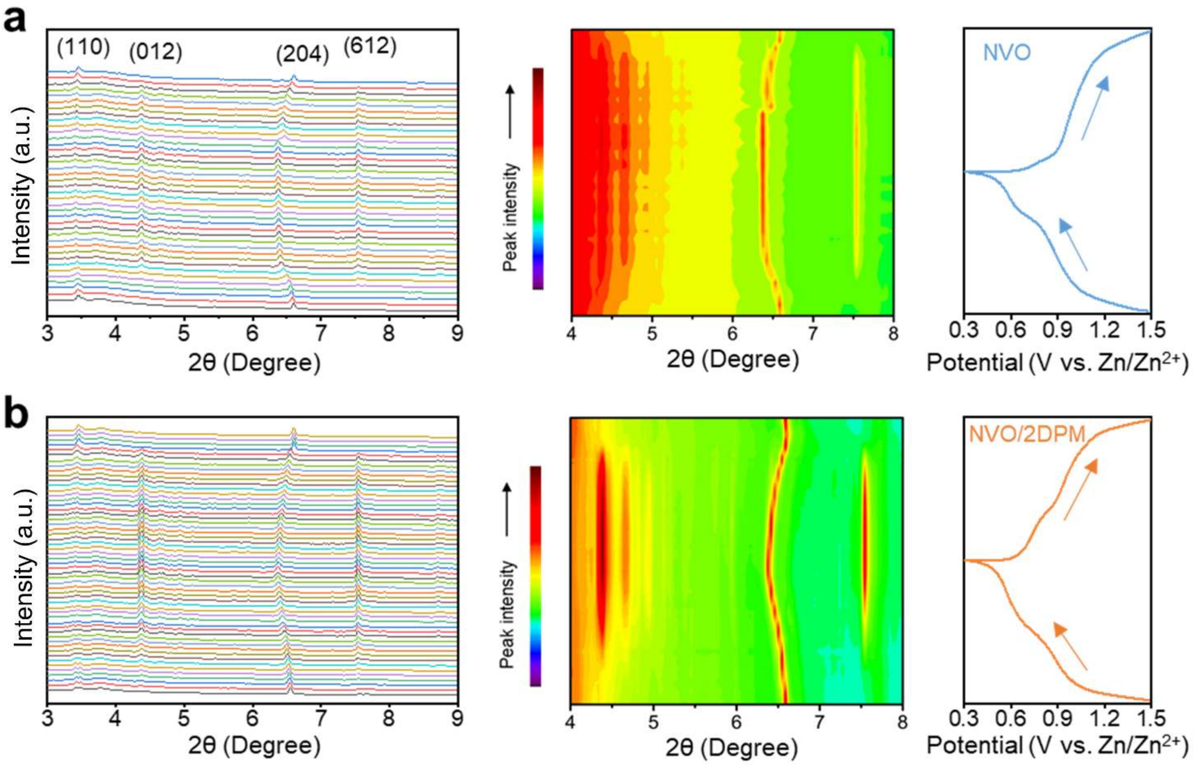

Supplementary Fig. 32. Operando synchrotron XRD. Operando synchrotron XRD patterns of **a** NVO and **b** NVO/2DPM. In both NVO and NVO/2DPM, ion insertion during the discharge step causes the appearance of new peaks (012, 612) corresponding to ion-inserted NVO. Meanwhile, continuously negative shift was observed for the (204) peak between 6.55 ~ 6.37°, which originates from the spacing expansion between V_3O_8 layers consists of edge-sharing VO_5 tetragonal pyramids and VO_6 octahedrons[5]. During the charge cycle, all peaks undergo the reversed change trend and recover to the initial state at 1.5 V vs. Zn/Zn^{2+}.

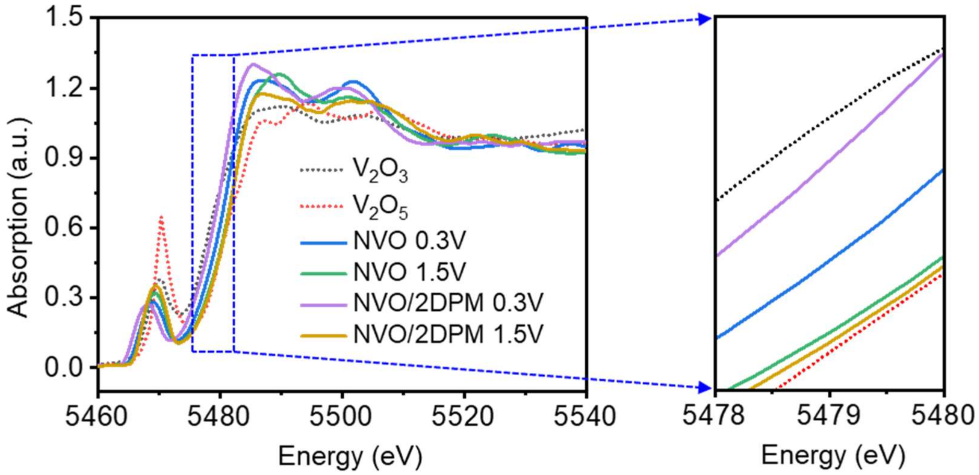

Supplementary Fig. 33. Normalized V K-edge X-ray absorption near edge structure of NVO and NVO/2DPM at fully charged and discharged states. The edge energy of fully charged NVO and fully charged NVO/2DPM is similar, implying their similar valence state close to V^{5+}. V K-edge of fully discharged NVO/2DPM shifts to an apparently lower energy than that of fully discharged NVO, reflecting the deeper redox depth of NVO/2DPM.

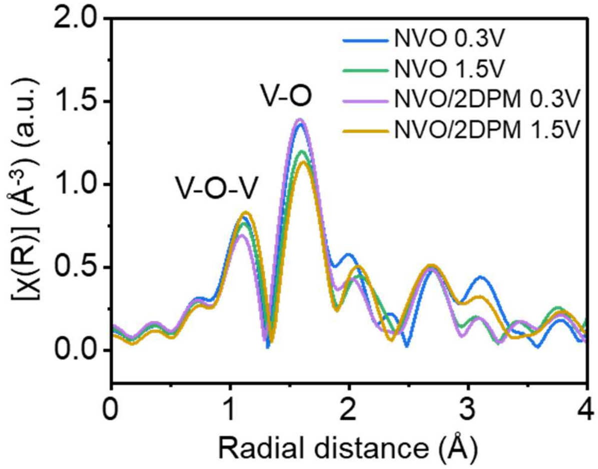

Supplementary Fig. 34. Fourier-transform of V K-edge extended X-ray absorption fine structure spectra (EXAFS) of NVO and NVO/2DPM at fully charged and discharged states. In all samples, the intensity maximum appears at around 1.6 Å in R space, corresponding to the first-shell V-O scattering. The wavelet-transform analysis of EXAFS in *R* and *k* spaces explicitly disclose the local coordination environment of V in NVO and NVO/2DPM.

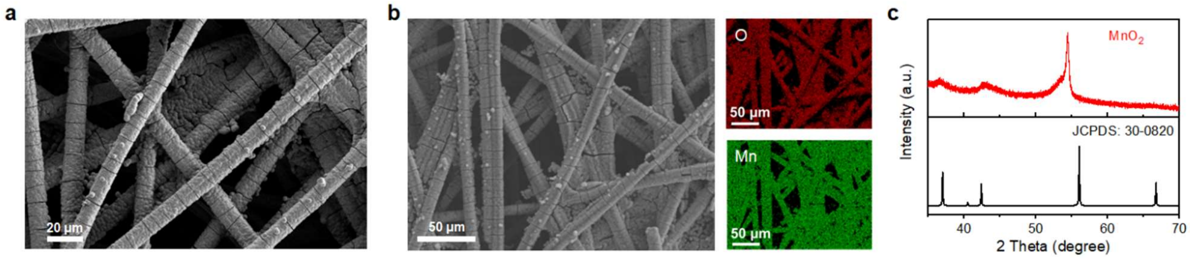

Supplementary Fig. 35. Structural characterizations of ε-MnO_2. a SEM image, **b** corresponding element mapping images, and **c** XRD pattern of ε-MnO_2 grown on carbon paper. The characteristic XRD peaks of ε-MnO_2 agree well with akhtenskite ε-MnO_2 (JCPDS card no. 30-0820)[27].

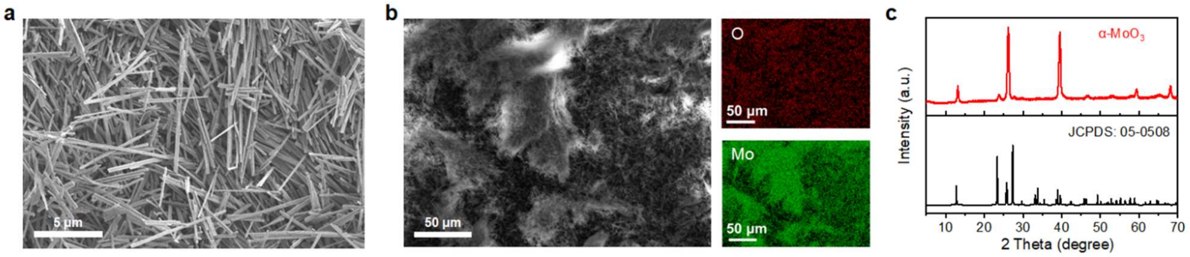

Supplementary Fig. 36. Structural characterizations of α-MoO_3. a SEM image, **b** corresponding element mapping images, and **c** XRD pattern of α-MoO_3 nanorods. The characteristic XRD peaks agree well with α-MoO_3 (JCPDS card no. 05-0508)[21].

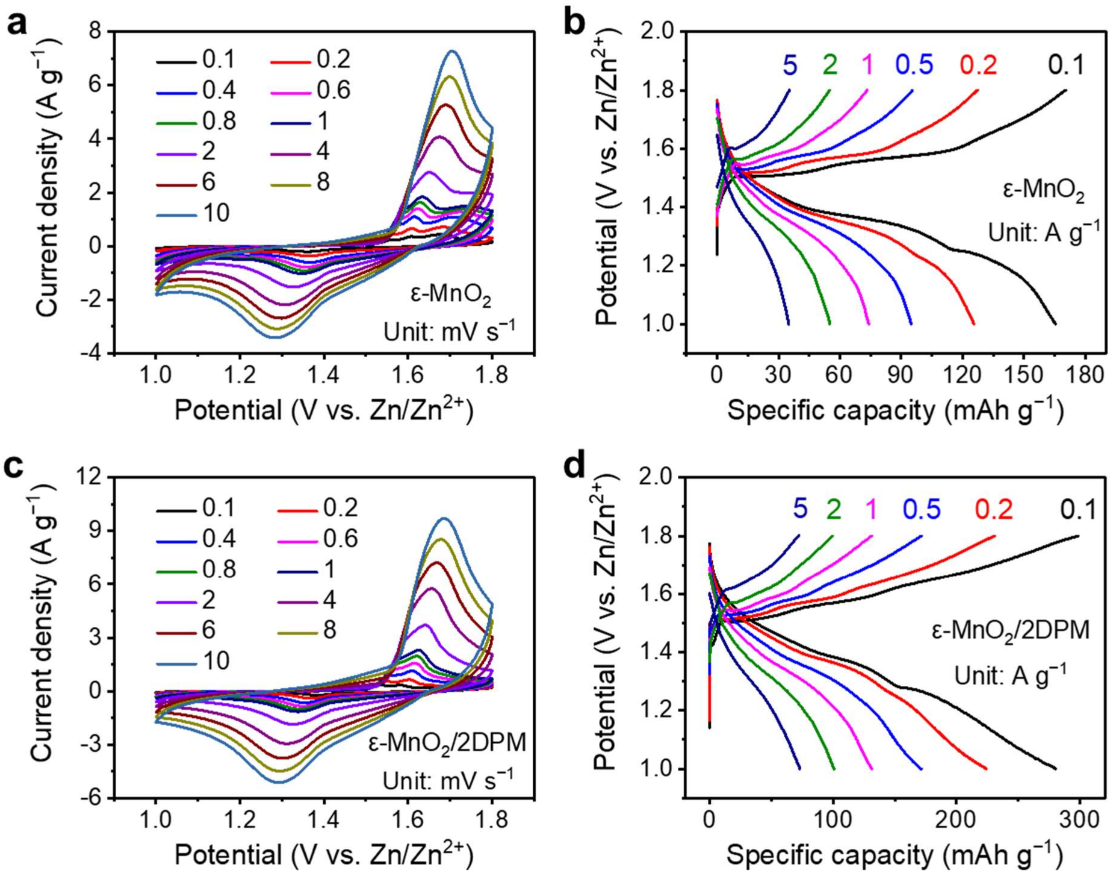

Supplementary Fig. 37. Electrochemical performance of ε-MnO_2 and ε-MnO_2/2DPM. a CV and **b** GCD curves of ε-MnO_2 in 2 M $ZnSO_4$. **c** CV and **d** GCD curves of ε-MnO_2/2DPM in 2 M $ZnSO_4$.

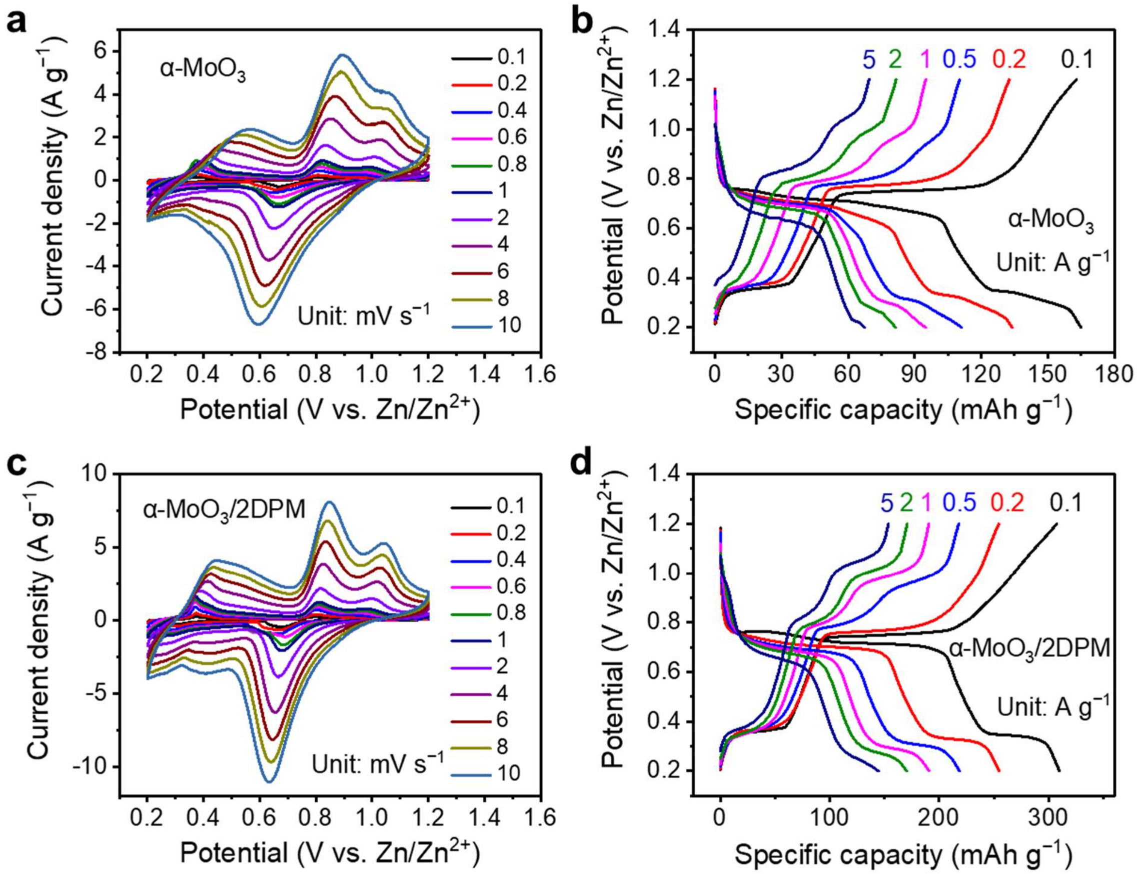

Supplementary Fig. 38. Electrochemical performance of α-MoO_3 and α-MoO_3/2DPM. a CV and **b** GCD curves of α-MoO_3 in 20 m $ZnCl_2$. **c** CV and **d** GCD curves of α-MoO_3/2DPM in 20 m $ZnCl_2$.

References

1 Sahabudeen, H. *et al.* Highly crystalline and semiconducting imine-based two-dimensional polymers enabled by interfacial synthesis. *Angew. Chem. Int. Ed.*. **59**, 6028-6036 (2020).

2 Zhang, Z. *et al.* Cation-selective two-dimensional polyimine membranes for high-performance osmotic energy conversion. *Nat. Commun.* **13**, 3935 (2022).

3 Zhou, Z. *et al.* Flexible ionic conjugated microporous polymer membranes for fast and selective ion transport. *Adv. Funct. Mater.* **32**, 2108672 (2021).

4 Mogg, L. *et al.* Perfect proton selectivity in ion transport through two-dimensional crystals. *Nat. Commun.* **10**, 4243 (2019).

5 Wan, F. *et al.* Aqueous rechargeable zinc/sodium vanadate batteries with enhanced performance from simultaneous insertion of dual carriers. *Nat. Commun.* **9**, 1656 (2018).

6 Luo, M. et al. Dynamic Regulation of the Interfacial pH for Highly Reversible Aqueous Zinc Ion Batteries. *Nano Lett.* **23**, 9491-9499 (2023).

7 Lin, C. et al. Adaptive Ionization-Induced Tunable Electric Double Layer for Practical Zn Metal Batteries over Wide pH and Temperature Ranges. *ACS Nano* **17**, 23181-23193 (2023).

8 Lyu, Y. et al. Organic pH Buffer for Dendrite-Free and Shuttle-Free Zn-I2 Batteries. *Angew. Chem. Inter. Ed.* **62**, e202303011 (2023).

9 Sauerbrey, G. Z. Use a quartz vibration form weigh thin films on a microbalance. *J. Phys.* **155**, 206-210 (1959).

10 Alfaruqi, M. H. *et al.* Electrochemical zinc intercalation in lithium vanadium oxide: a high-capacity zinc-Ion battery cathode. *Chem. Mater.* **29**, 1684-1694 (2017).

11 Xia, C. *et al.* Highly stable aqueous zinc-Ion storage using a layered calcium vanadium oxide bronze cathode. *Angew. Chem. Int. Ed. Engl.* **57**, 3943-3948 (2018).

12 Kundu, D. *et al.* A high-capacity and long-life aqueous rechargeable zinc battery using a metal oxide intercalation cathode. *Nat. Energy* **1**, 16119 (2016).

13 Xia, C. *et al.* Rechargeable aqueous zinc-ion battery based on porous framework zinc pyrovanadate intercalation cathode. *Adv. Mater.* **30**, 1705580 (2018).

14 Li, J. *et al.* Multi-scale investigations of δ-$Ni_{0.25}V_2O_5 \cdot nH_2O$ cathode materials in aqueous zinc-ion batteries. *Adv. Energy Mater.* **10**, 2000058 (2020).

15 Wan, F. *et al.* Reversible oxygen redox chemistry in aqueous zinc-Ion batteries. *Angew Chem. Int. Ed. Engl.* **58**, 7062-7067 (2019).

16 Pan, H. *et al.* Reversible aqueous zinc/manganese oxide energy storage from conversion reactions. *Nat. Energy* **1**, 16039 (2016).

17 Zhang, N. *et al.* Rechargeable aqueous zinc-manganese dioxide batteries with high energy and power densities. *Nat. Commun.* **8**, 405 (2017).

18 Huang, J. *et al.* Polyaniline-intercalated manganese dioxide nanolayers as a high-performance cathode material for an aqueous zinc-ion battery. *Nat. Commun.* **9**, 2906 (2018).

19 Guo, S. *et al.* Cathode interfacial layer formation via in situ electrochemically charging in aqueous zinc-Ion battery. *ACS Nano* **13**, 13456-13464 (2019).

20 Zhang, N. *et al.* Cation-deficient spinel $ZnMn_2O_4$ cathode in $Zn(CF_3SO_3)_2$ electrolyte for rechargeable aqueous Zn-ion battery. *J. Am. Chem. Soc.* **138**, 12894-12901 (2016).

21 Yu, M. *et al.* Interlayer gap widened alpha-phase molybdenum trioxide as high-rate anodes for dual-ion-intercalation energy storage devices. *Nat. Commun.* **11**, 1348 (2020).

22 Zhang, H. *et al.* Interlayer engineering of α-MoO_3 modulates selective hydronium intercalation in neutral aqueous electrolyte. *Angew. Chem. Int. Ed. Engl.* **60**, 896-903 (2021).

23 Li, H. *et al.* MoS_2 nanosheets with expanded interlayer spacing for rechargeable aqueous Zn-ion batteries. *Energy Stor. Mater.* **19**, 94-101 (2019).

24 Zhang, L., Chen, L., Zhou, X. & Liu, Z. Towards high-voltage aqueous metal-Ion batteries beyond 1.5 V: the zinc/zinc hexacyanoferrate system. *Adv. Energy Mater.* **5**, 1400930 (2015).

25 Zeng, Y. *et al.* Construction of Co-Mn Prussian blue analog hollow spheres for efficient aqueous Zn-ion batteries. *Angew. Chem. Int. Ed. Engl.* **60**, 22189-22194 (2021).

26 Zhang, P. et al. Dual-redox-sites enable two-dimensional conjugated metal-organic frameworks with large pseudocapacitance and wide potential window. *J. Am. Chem. Soc.* **143**, 10168-10176 (2021).

27 Sun, W. *et al.* Zn/MnO_2 battery chemistry with H^+ and Zn^{2+} coinsertion. *J. Am. Chem. Soc.* **139**, 9775-9778 (2017).
